# The Effect of Ultrasound on the Extraction and Functionality of Proteins from Duckweed (*Lemna minor*)

**DOI:** 10.3390/molecules29051122

**Published:** 2024-03-01

**Authors:** Vicente Antonio Mirón-Mérida, Cintya Soria-Hernández, Alejandro Richards-Chávez, Juan Carlos Ochoa-García, Jorge Luis Rodríguez-López, Cristina Chuck-Hernández

**Affiliations:** 1 Escuela de Ingeniería y Ciencias, Tecnologico de Monterrey, Av. Eugenio Garza Sada 2501, Colonia Tecnológico, Monterrey 64700, Mexico; vicente.miron.m@tec.mx (V.A.M.-M.); a01632538@tec.mx (A.R.-C.); a00832933@tec.mx (J.C.O.-G.); a00831992@tec.mx (J.L.R.-L.); 2 Instituto para la Investigación en Obesidad, Tecnologico de Monterrey, Av. Eugenio Garza Sada 2501, Sur Tecnológico, Monterrey 64849, Mexico

**Keywords:** duckweed, ultrasound-assisted extraction, protein, design of experiments, functional properties

## Abstract

The inclusion of protein in the regular human diet is important for the prevention of several chronic diseases. In the search for novel alternative protein sources, plant-based proteins are widely explored from a sustainable and ecological point of view. Duckweed (*Lemna minor*), also known as water lentil, is an aquatic plant with potential applications for human consumption due to its protein content and carbohydrate contents. Among all the conventional and novel protein extraction methods, the utilization of ultrasound has attracted the attention of scientists because of its effects on improving protein extraction and its functionalities. In this work, a Box–Behnken experimental design was proposed to optimize the alkaline extraction of protein from duckweed. In addition, an exploration of the effects of ultrasound on the morphological, structural, and functional properties of the extracted protein was also addressed. The optimal extraction parameters were a pH of 11.5 and an ultrasound amplitude and processing time of 60% and 20 min, respectively. These process conditions doubled the protein content extracted in comparison to the value from the initial duckweed sample. Furthermore, the application of ultrasound during the extraction of protein generated changes in the FTIR spectra, color, and structure of the duckweed protein, which resulted in improvements in its solubility, emulsifying properties, and foaming capacity.

## 1. Introduction

Proteins are biomacromolecules that have been part of several diets across the world, yet their role in modulating eating patterns towards preventing obesity, diabetes, and other chronic diseases [1,2] has increased interest in exploring different protein sources and their potential applications. Furthermore, the type of consumed protein plays a relevant role in the impact of such molecules on overall health. In this regard, when compared to animal proteins, the consumption of plant-based protein has been associated with a greater muscle mass in diabetic and elderly patients as well as a lower risk of developing cardiovascular diseases, type 2 diabetes, unhealthy aging, and malnutrition, hence a lower mortality risk [3,4,5,6,7,8,9,10].

In addition to the beneficial effects of plant proteins on preventing cardiometabolic and other diseases [11], these have been reported as a more sustainable alternative to animal proteins by means of their reduced generation of greenhouse gases and a more positive impact on the environment [12,13]. Among the diverse alternative proteins that are currently utilized and explored, duckweed comprises a group of five genera of floating aquatic plants from the *Lemnaceae* family (*Landoltia*, *Lemna*, *Spirodela*, *Wolffian*, and *Wolffiella*) [14,15,16,17] that have emerged as an option for the exploration of its potential use as a food protein source. Duckweeds have been used as feed replacements for livestock and fish [15,18], as well as a source of secondary metabolites with medicinal properties [16]. Nevertheless, the rising interest in duckweed as a potential nutritious food matrix relies on its amino acid and vitamin absorption and availability [15]. Similarly, the use of duckweed in the bioremediation of wastewater results in the production of biomass with promising protein and starch contents [17].

Our of the several protein extraction methods, the utilization of ultrasound (US) confers a green and clean label option by means of its optimized extraction times and yields in combination with lower energy demands [19,20]. In general, the principle of ultrasound consists of the direct or indirect emission of waves into a sample [20], where the cavitation effect generates the formation of bubbles within the liquid media, which upon implosion generate energy (temperature, pressure) increments, resulting in cell disruption [19,20]. Regardless of the utilized ultrasound system, there are physical parameters that are relevant when optimizing the application of this method, namely the ultrasound frequency (20–100 kHz), power, intensity, amplitude, power density, and cycles (continuous or pulses) [20], which in some cases can be manually controlled on the instrument. The ultrasound-assisted extraction of proteins has been studied due to its enhanced mass transfer and extract yields [19], where the medium and matrix properties are important for the maximized extraction of proteins. For example, the solid/solvent ratio, particle size, and selected solvent are essential for improving extraction yields [20,21]. Moreover, the extraction process is helped by the temperature increments produced by ultrasound, which promote the diffusion of molecules and the release of matrix-bound compounds, yet an uncontrolled temperature increment could lead to lower protein yields as well as lower intensities achieved by the implosion of microbubbles [20].

Furthermore, special interest has been focused on the effect of ultrasound on the functional properties of proteins, as denoted by the improved solubility and water and oil holding capacity, as well as the formation of emulsions and dispersion of already extracted proteins [22,23]. Similarly, ultrasound can also modify the molecular structure and size of proteins, which has an impact on the droplet size of emulsions [24]. Many of the effects conferred by ultrasound are mediated and depend on the ultrasound conditions and the protein type [25]. As noted, the functional or techno-functional properties of food proteins are diverse, as they describe the way in which proteins physically and chemically interact with water, oil, and air, which is mainly attributed to the amphiphilic structure of peptides within the proteins. Based on this, the techno-functional properties of proteins include solubility, water and oil-holding capacity, as well as foaming, emulsifying, and gelling proteins [24,25].

Apart from the effects of ultrasound on extracted proteins, the integration of ultrasound to assist conventional extraction methods has been considered for plant-based proteins, as they are commonly bound to carbohydrates and secondary metabolites. Compared to conventional extraction methods, alkali extraction is broadly utilized due to its advantages in terms of cost, digestibility, and bioavailability [26]. In this method, the proteins are solubilized in basic conditions (pH 8.5–9) through the ionization of amino acids, rupture of disulfide bonds, and an overall negative surface charge, followed by a protein precipitation step at the isoelectric point (pH ~4.5) and a collection or a neutral solubilization [20,21,26]. Nevertheless, a high pH has been associated with protein denaturation, color modification, and a reduced purity and protein yield equivalent to 50% of the extractable proteins [20,21,26].

For those reasons, the alkaline extraction of proteins can be assisted with ultrasound, as this combination could release more protein through cell disruption and a reduction in the sample’s particle size, whose effects are expected in the functional properties of the resulting protein extracts and isolates [21,26]. In the case of alkaline extraction assisted by ultrasound, the extraction time is another parameter that must be controlled to avoid protein aggregation, a reduction in protein yield, and changes in the tertiary and secondary structures of protein that might be caused by prolonged exposure to heat and shear [20,21]. It should be noted that the changes in the secondary structure generated during the ultrasound-assisted extraction step could improve the functionality of proteins [27,28]. Moreover, the involvement of different forces makes ultrasound an attractive option for improving extraction yield [27].

Hence, in this research, the optimization of the ultrasound-assisted extraction of protein from duckweed (*Lemna minor*) was carried out by modifying the dispersion pH and the ultrasound time and amplitude. Additionally, a study of the effect of ultrasound on the structural, morphological, and functional properties of the protein extracts was performed by comparing them with a control sample obtained from an alkaline extraction without the integration of ultrasound. This work reports this type of comparison for a specific duckweed subspecies (*Lemna minor*), with particular exploration of high alkaline conditions.

## 2. Results and Discussion

### 2.1. Proximate Analysis of Duckweed (Lemna minor)

The proximate analysis of duckweed flour (*Lemna minor*) is presented in Table 1 [29,30,31,32,33,34,35,36], where a comparison with the reported values for other duckweed species is made. The main difference among species was observed in the moisture content; however, this was expected, as each sample was either analyzed fresh [29,31] or after a drying treatment [33,34,36].

Although in all the species the main nutrients were carbohydrates, proteins, and ashes, intraspecies differences in the proximate composition of duckweed are expected due to cell physiology and morphological variations [14,37]. The nutritional composition of duckweed also depends on the growth conditions and is mainly related to the medium components [33]. Additionally, the protein content is also dependent on the species and the extraction method [14]. The amount of protein found (21.74%) in this work corresponds to the reported values for *Lemna minor* [16], and duckweed protein is promising for protein consumption because of the provision of all the essential amino acids, relatively good digestibility, and bioavailability, with potential applications in the dairy and beverage industries [14].

An important aspect to be considered towards the application of duckweed protein for human consumption is the presence of undesired compounds and molecules in the medium. As many bodies of water are highly polluted, the presence of heavy metals, toxins, and pesticides is a concern. In addition, some undesired antinutrients, such as oxalic acid and phytates, might limit the assimilation of nutrients from duckweed. Nevertheless, some metabolites with benefits to health, namely alkaloids, flavonoids, and saponins, can also be found in duckweed [36]. Therefore, special attention should be given to the growth conditions of duckweed, as the presence of certain nutrients along with the pH conditions could result in the accumulation of nitrate and their derivatives, along with the aforementioned molecules and elements [14,38], if present in the growth media.

### 2.2. Optimization of the Extraction of Protein from Duckweed

All the factor combinations explored in the Box–Behnken experimental design, along with their corresponding responses, are displayed in Table 2. The optimal point was found at a pH of 11.5 and an ultrasound amplitude and processing time of 60% and 20 min, respectively. After the experimental design analysis, the significance of each factor and combination was obtained using an analysis of variance (Tables S1–S3) and pareto diagrams (Figures S1–S3). As noted, pH was the most significant (*p* < 0.05) factor for all the responses. Apart from this, the ultrasound time and its combination with the pH were significant (*p* < 0.05) for the solids yield (SY), while the combination of pH and amplitude was also significant (*p* = 0.047) for the protein extraction yield (PEY).

**Figure 1:**
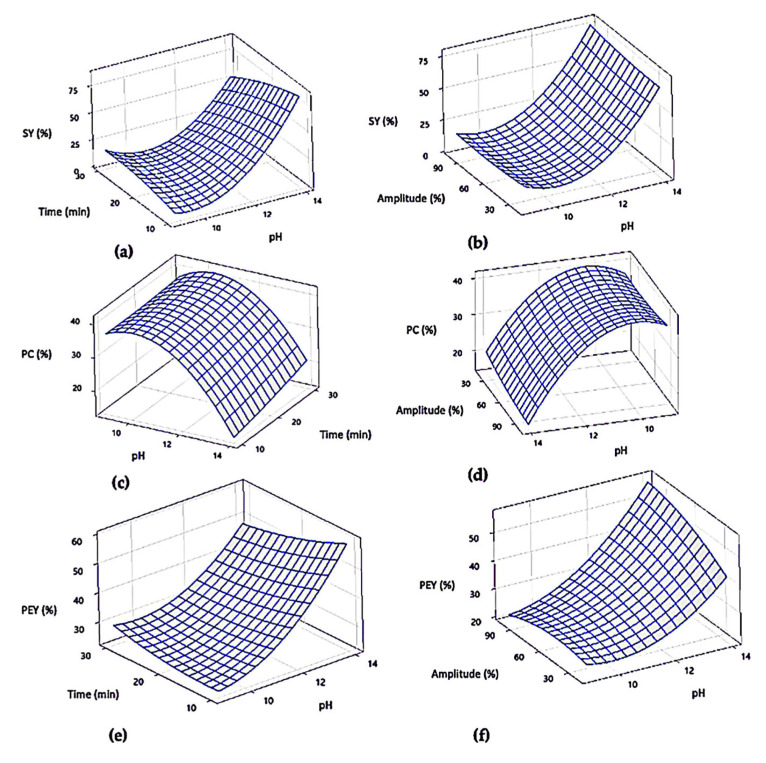
Response surface of the effects of pH with the (**a**) ultrasound time and (**b**) amplitude on the solids yield (SY, fixed amplitude = 60%, and fixed time = 20 min); (**c**) ultrasound time and (**d**) amplitude on the protein content (PC, fixed amplitude = 60%, and fixed time = 20 min); (**e**) ultrasound time and (**f**) amplitude on the protein extract yield (PEY, fixed amplitude = 60%, and fixed time = 20 min).

The uncoded mathematical models for each response are indicated in Table 3, where quadratic equations were obtained to describe the variability of data. Each model was a good predictor, given by their determination coefficients (r^2^). Hence, based on these values, surface plots were obtained for each response (Figure 1). As the pH was the only significant value for the three responses, the surface plots for each response show the combination of this factor with both the amplitude and the ultrasound time, while the excluded factor was kept at the optimal value (amplitude = 60% or time = 20 min). As indicated in Figure 1a,b, a higher SY is obtained with a high pH value (~14) in combination with 10–20 min of ultrasound. As previously stated, the amplitude is not significant for the SY (*p* = 0.535), so a high pH is necessary for an increase in the SY values, regardless of the ultrasound amplitude value. In accordance with the observed significance in this work, previous optimization studies for the extraction of protein from duckweed have denoted the significant effect of pH (*p* = 0.0007), which, in combination with greater extraction temperatures (80 °C), resulted in more protein solubilization [33].

The surface responses for the protein content (PC) are shown in Figure 1c,d, where it can be noted that a pH close to 11–11.5 is required for a high PC despite the ultrasound time and amplitude, which is expected as the pH was the only significant factor for this response (*p* < 0.05). On the other hand, the surface responses for the PEY in Figure 1e,f denote the need for higher pH values with a low ultrasound time and amplitude for a greater protein yield, yet a more notorious effect is observed between the amplitude and pH values, which is the only significant factor combination for the PEY. In this regard, different optimal points reported for duckweed demonstrated the need for 25 min of extraction, an US amplitude of 78% and a pH of 11 for achieving the highest protein solubility during an extraction, and a PEY value [33,39], which is consistent with the optimal point found in this work (pH = 11.5, US time = 20 min, and US amplitude = 60%).

For the validation of the experimental design, the optimal point was predicted in Minitab and replicated in the laboratory, and the results are reported in Table 4. This point allowed a correct validation based on the overall better predicted and real values for the three responses (compared to other treatments), as well as their good fit between both values. It is worth mentioning that, as not all the factors and their combinations were significant for each response, this was selected as the optimal point, as targeting the maximization of a single response diminished the outcome of the other untargeted responses. Likewise, the condition for maximizing the three responses (pH = 13.4, amplitude 21.61%, time 10 min) was not considered for validation because of the lower predicted PC value, which was similar to the original duckweed flour (21.74%), due to the combination of three different models at a time (predicted values: SY = 64.75%, PC = 23.84%, PEY = 45.20%).

In the US-assisted extraction of protein, a high amplitude, in which more bubbles are collapsing from cavitation, brings a higher power and intensity of waves, resulting in a higher SY by means of more plant cell disruption than from the sole extraction in alkaline conditions [20,21]. Nevertheless, a high amplitude is also related to foam formation and diminished ultrasound performance [21], which was not the case for some of the lower PC values obtained at a high amplitude (Table 1), mainly attributed to the changes in pH. Additionally, a longer ultrasound time represents a temperature increase in the sample, with a high risk of protein denaturation [26]; for that reason, an ice bath and 5 min cycles (pulses) were integrated with the ultrasound system.

Alkaline conditions are utilized to break the bonds between the targeted proteins and the food matrix components, namely, interactions between hydrogen, carboxylic, and sulphate ions. This condition must be optimized to avoid protein denaturation, and even when a pH of 6.8–7 has been reported as optimal for extracting plant-based proteins, the combination of high pH and ultrasound was optimal for achieving greater SYs, as a maximum pH value needs to be established for each specific sample to prevent any decrease in the protein yield [20].

Because of the above, an exploration of high alkaline conditions was performed in this work, where, for optimization purposes, a pH of 11.5 in combination with an amplitude of 60 and 20 min under ultrasound was selected as the optimal point. This point does not apply to the highest levels for each condition because, when increasing the temperature (amplitude) and processing time, a protein extraction plateau is reached, from which the right combination of time, amplitude, and pH is proposed to avoid energy consumption and higher costs upon scaling up [20].

As demonstrated in Table 2 and Table 4, despite the extraction optimization, the obtained PEY values denote the challenges when extracting RuBisCo and other green leaf proteins, as their location within the leaf matrix in combination with the high moisture content of leaves limits the obtention of high protein yields and pure protein isolates, as well as their industrial exploitation and applicability [29,40].

### 2.3. Evaluation of the Effect of Ultrasound on the Alkaline Extraction of Protein from Duckweed (Lemna minor)

To evaluate the effect of ultrasound on the alkaline extraction of proteins, the optimal point (pH = 11.6, US amplitude = 60%, and US time = 20 min) was reproduced, and the extracted protein was collected by precipitation at pH 4.5. Moreover, a control sample was produced by following the same steps, alkalinity, ratios, and volumes, without the integration of the ultrasound treatment before incubating. The protein content of the ultrasound protein extract (40.43 ± 0.90% wb) was significantly greater (*p* < 0.05) than the values quantified in the control protein extract (31.69 ± 1.67% wb) and was double the value for duckweed (20.38 ± 0.29% wb); hence, more protein was extracted with the aid of ultrasound. A central composite optimization for the protein extraction in duckweed denoted a higher protein solubilization at pH values above 11, as more charged proteins allow their interaction with water. At this pH, RuBisCO, the carbon-fixing protein commonly reported in duckweed, is separated into its subunits, which can be unfolded with a temperature increase [29,33]. In addition, a combination of high ultrasound intensity and short time is always prioritized in plant-based proteins to avoid denaturation, functionality losses, and additional costs [26].

#### 2.3.1. Morphological and Structural Effects of Ultrasound on the Extracted Protein

The color parameters (L*, a*, and b*) of duckweed and its derived control and ultrasound protein extracts are indicated in Table 5. The L* and a* values between the duckweed flour and the control protein extract had no significant differences, while the ultrasound protein extract had significantly lower lightness (L*) and higher a* and b* values, which are related to more redness and yellowness in the sample [41]. Similar results were already observed in biodegradable plastics made from duckweed biomass mixed with polylactic acid and cassava starch, where the addition of more duckweed biomass resulted in lower lightness and a resulting color in the red and yellow regions [42]. The appearance of each sample portrayed different intensities of green, where the ultrasound protein extract had the darkest intensity, which corresponds with a lower L* value. Nevertheless, the samples were similar to those reported for duckweed flour and its corresponding protein materials, where the green off-color was related to chlorophyll a/b binding proteins as well as chlorophyll [33]. For this reason, some methods, such as mild coagulation, are sometimes included to eliminate the green color in the protein derivatives from duckweed [29], yet ultrasound-assisted extraction is also utilized for the extraction of chlorophyll and other pigments of interest from plants [43].

Apart from RuBisCo, one of the most common protein types found in the chloroplasts of leaves are chlorophyll light-binding complexes [40]. Furthermore, the transformation of chlorophyll into pheophytin by acidic conditions results in an olive-green-colored product. Those changes can be prevented by alkaline conditions in which chlorophylls are maintained [44]. In addition, the integration of ultrasound might be beneficial for extracting not only chlorophyll but also carotenoids, which can also be found in the chloroplasts of duckweed to support photosynthesis [40,45,46].

**Figure 2:**
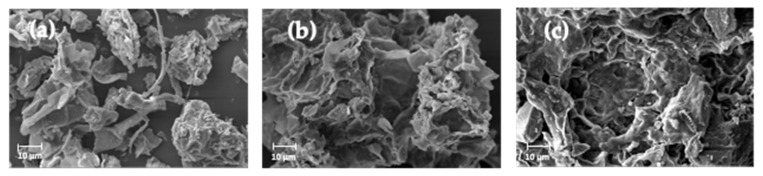
SEM images for (**a**) duckweed flour, (**b**) control protein extract, and (**c**) ultrasound protein extract at a magnification of 1000×.

The SEM images of duckweed flour and its corresponding control and ultrasound protein extracts are displayed in Figure 2. The morphology of duckweed shows uneven particles of different sizes, whose irregular surfaces have been previously reported in this type of material [42] and were expected due to the possible effect of sun-drying on the morphology of the duckweed flour. In the case of the protein extract assisted by ultrasound, a network of open spaces was observed within the sample matrix as ultrasound generated cell rupture and a fragmented structure, where the disrupted cell components appeared aggregated [21]. Those spaces, also referred to as perforations, are an indication of breakdown from ultrasound [47]. The observed swelling and aggregation observed in the protein extract can be related to the application of alkaline conditions to the duckweed matrix [48].

**Figure 3:**
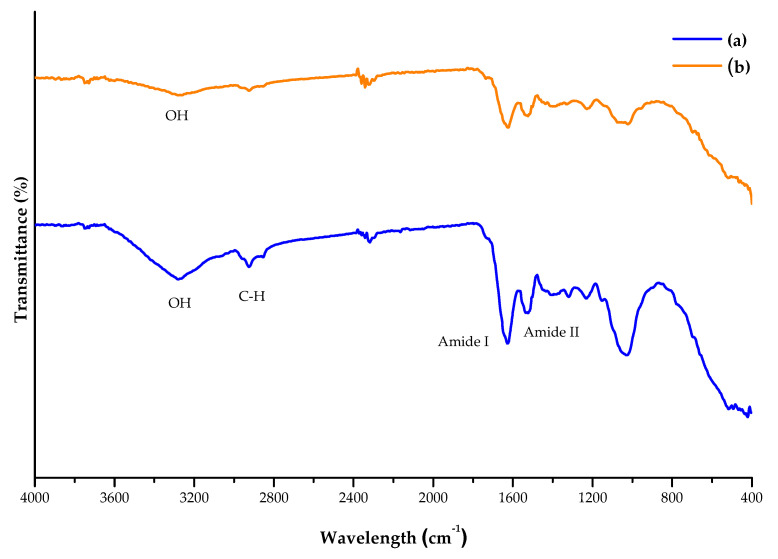
FTIR spectra for the (a) control and (b) ultrasound protein extracts from duckweed between 4000 and 400 cm^−1^.

The FTIR spectra for the control protein sample and the ultrasound protein sample are displayed in Figure 3, where different regions correspond to certain molecules and structural changes produced by ultrasound are noticed. The pronounced O-H stretching (3600 to 3200 cm^−1^) region is associated with either water or carbohydrates, while the 3000 to 2800 cm^−1^ region, especially found in the control sample, is attributed to C-H stretching, as well as CH_2_ and CH_3_ from macromolecules such as fats, carbohydrates, and proteins [33]. Likewise, the 1750–1700 cm^−1^ region corresponds to the C=O stretching from lipids and is an indicator of their oxidation degree [49]. The bands at 1000 and 1100 cm^−1^ are, respectively, related to the presence of starch and carbohydrates in the sample [49], which other authors considered between 1250 and 800 cm^−1^ [33].

Regarding proteins, the bands at 3400, 2950, and 1300 cm^−1^ correspond to amides A, B, and III, respectively. Moreover, amide I was found at 1650 cm^−1^ due to peptide C=O stretching, while amide II can be found at the 1550 to 1430 cm^−1^ region because of vibrations from NH bending and CN stretching [50]. Based on previous reports, the amide I band (~1610 cm^−1^) indicates the secondary structure, which in this case is mainly associated with inter and intramolecular parallel and antiparallel β-sheets [33,50] than other secondary structures. The main differences between the ultrasound and control samples were found in the 2926–2924 and 1032–1026 and cm^−1^ regions, which were more pronounced in the control sample, indicating the presence of more carbohydrates [33].

**Figure 4:**
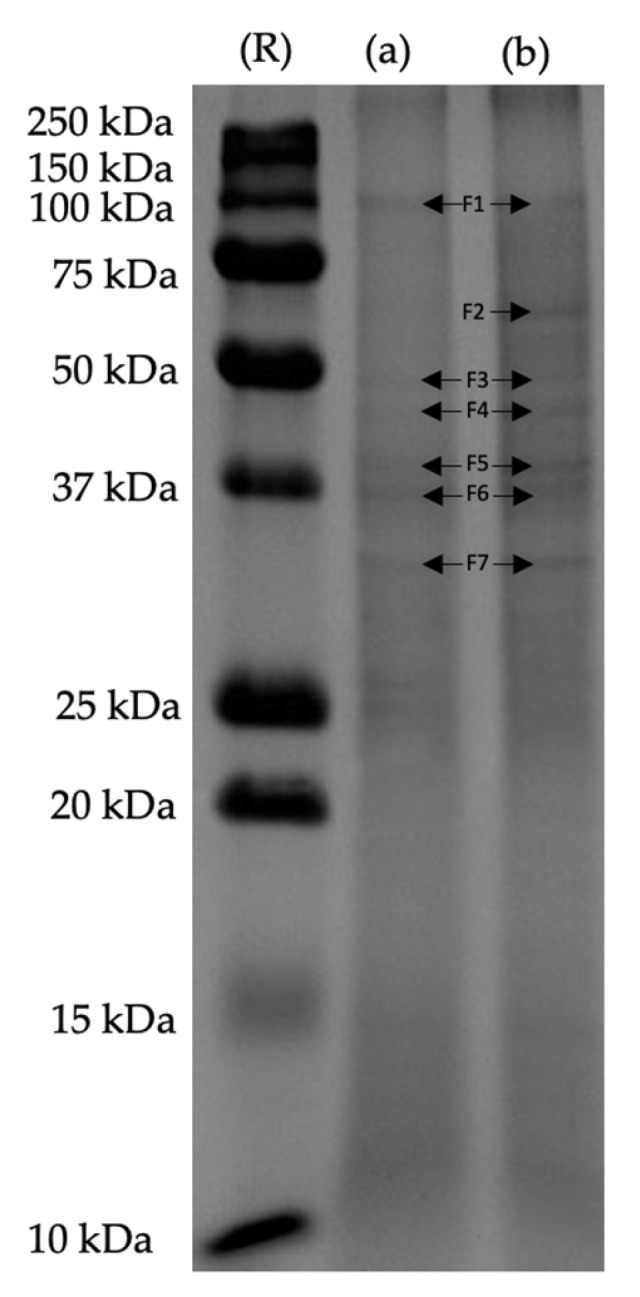
Molecular weight profile of the (**a**) control and (**b**) ultrasound protein extracts from duckweed by SDS-PAGE. Reference (**R**); 100 KDa fraction (F1); 63 KDa fraction (F2); 50 KDa fraction (F3); 45 KDa fraction (F4); 39 KDa fraction (F5); 37 KDa fraction (F6); and 32 KDa fraction (F7).

Figure 4 shows the molecular weight profiles of the control and ultrasound protein extracts from duckweed (*Lemna minor*). Seven protein fractions of 100, 62.5, 50, 45, 39, 37, and 32 kDa were observed in the ultrasound sample, while six protein fractions with lower intensities were identified in the control sample. This confirms that the application of ultrasound was beneficial for extracting more proteins from duckweed.

An analysis of ambient dried, frozen, and fresh *Spirodela polyrhiza* demonstrated the presence of four bands with a minimum molecular weight of 14 kDa and the largest proteins found above 160 kDa [50]. A protein extract from *Wolffia globosa* obtained through ultrasound-assisted extraction (120 kHz, 15 min) had five main bands at 25, 30, 45, 63, and 100 kDa [34]; these values also corresponded with the bands reported at 20, 25, 30.6, 45.6, and 61.5 kDa for enzymatically prepared protein hydrolysates from the same duckweed species [39]. The bands at 14 and 56 kDa can be attributed to subunits of RuBisCo [40,51], which have been fully observed in *Spirodela polyrhiza* [50] and in *Lemna minor* as an abundant band between 50 and 60 kDa [52].

Although the impact of ultrasound for extracting specific proteins from *Lemna minor* and other duckweed species has not been reported, it has been confirmed that ultrasound can increase the protein yield from *Lemna minor*; however, long exposure to cavitation could result in the denaturation of soluble proteins along with the extraction of more chlorophyll [47], which corresponded with the color of the control and ultrasound samples, as well as the observed background in their SDS-PAGE lanes. Other molecules such as fatty acids and secondary metabolites (phenolics) can interfere with the extraction of proteins; therefore, different extraction methods can result in diverse profiles of extractable proteins [53]. Based on the SDS-PAGE result, we observed a beneficial effect from ultrasound on the extraction of protein fractions that are difficult to extract, as already reported for RuBisCo, which has been reported as the main protein in duckweed, regardless of its extraction limitations due to its location within the plant matrix. Hence, higher RuBisCo yields can be obtained due to the enhanced mass transfer produced by ultrasound cavitation [29,40]. This corresponds with the presence of a more intense band at 50 kDa in the ultrasound sample, as well as a unique band at 62 KDa.

#### 2.3.2. Functional Properties of the Duckweed Flour and Its Extracted Protein

**Figure 5:**
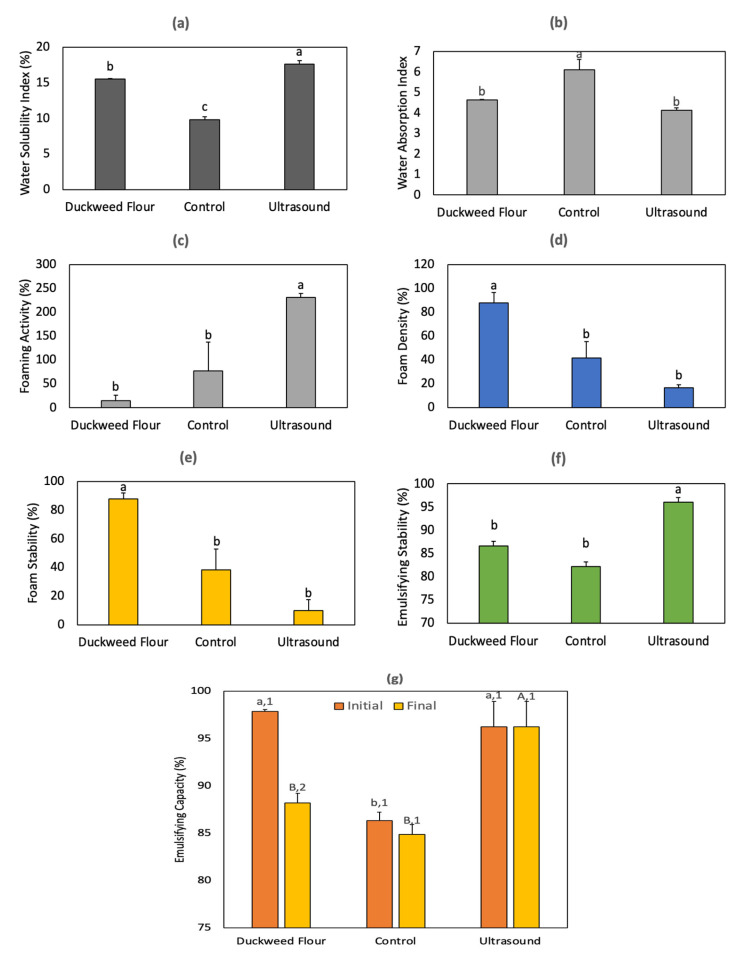
Functional properties of duckweed flour and control and ultrasound protein extracts including (**a**) water solubility index, (**b**) water absorption index, (**c**) foaming activity, (**d**) foam density, (**e**) foam stability, (**f**) emulsion stability (different letters indicate significant differences between samples), and (**g**) initial and final (after 24 h) emulsifying capacity (lowercase and uppercase letters, respectively, indicate significant differences for the initial and final values between different samples, while different numbers indicate significant differences between the initial and final values of the same sample). Experimental data provided as mean (n = 3) with its corresponding standard deviation bar.

The functional properties of the three samples are indicated in Figure 5. As noted in Figure 5a, the ultrasound-assisted extraction of protein from duckweed was beneficial for acquiring a significantly different (*p* < 0.05) water solubility (17.62%), which in the case of the ultrasound sample represented an increase of 13.69% and 79.05% compared to the solubility for duckweed flour (15.49%) and the control extract (9.84%), respectively. On the contrary, the water absorption index (Figure 5b) of the control sample (6.11) was significantly higher (*p* < 0.013) than the values for duckweed flour (4.12) and the ultrasound sample (4.64). In the case of the water solubility index, a reduction in the particle size of aggregates produced by ultrasound allows more interactions with water, thus a higher solubility [24,26]. This is also produced by ultrasound through protein unfolding and exposure of its hydrophilic groups [25].

As shown in Figure 5c, the foaming activity of the ultrasound protein extract (230.89%) had a significant increase (*p* = 0.003) of 15-fold and 2-fold from the values for duckweed flour (14.52%) and the control protein extract (77.31%), respectively. Although the ultrasound protein extract had the best capacity to foam, the obtained volume had the lowest stability, as denoted in Figure 5e, where the obtained value (9.89%) represented an 8-fold reduction compared to the stability of duckweed flour (87.81%). Furthermore, the foam density was significantly higher (*p* = 0.002) for duckweed flour (87.76%), in comparison to both the control (41.71%) and ultrasound (16.52%) samples (Figure 5d).

In the case of emulsifying stability (Figure 5f), the emulsion produced with the ultrasound sample was significantly (*p* = 0.002) more stable (96.04%) than the emulsions with duckweed flour (86.64%) and the control sample (82.16%). Moreover, the initial emulsifying capacity of duckweed flour (97.89%) and ultrasound (96.24%) was comparably higher than the capacity of the control sample (86.33%); yet, after 24 h, only the ultrasound sample maintained its original emulsifying capacity (96.24%) with a significantly greater value (*p* = 0.002) than those from the emulsions with duckweed flour (88.22%) and control sample (84.86%).

The higher emulsifying and foaming activities correspond to the increased surface area and protein denaturation produced by the ultrasound-assisted extraction of proteins in combination with the alkaline conditions. Nevertheless, a reduced foaming capacity can occur due to the formation of aggregates [25]. The foaming activity of the control (77.31%) and ultrasound (230.90%) protein extracts from duckweed (*Lemna minor*, Figure 5c) was higher than the reported values for *Wolffia globosa* (35–55.5%). Unfortunately, the resulting foam stability (Figure 5e) for the ultrasound sample (9.89%) was lower than the initial duckweed flour (87.81%) and its control protein sample (38.48%), which in fact could be more suitable for possible applications when compared to *Wolffia globosa* (21–33%) [39]. Nevertheless, previously reported foaming capacity values (194–278%) for duckweed protein fractions, possibly from a different species to *Wolffia globosa*, were higher than the value for egg white (122%), yet the stability also decreased in all the duckweed samples [33], similar to what was observed in this work.

Likewise, the emulsifying capacity of duckweed (97.88%) and the ultrasound sample (96.24%, Figure 5g) were comparable to the reported values for *Wolffia globosa* (96–100%) [39]. Although the emulsifying stability (Figure 5f) of the ultrasound sample (96.05%) was higher than the observed values (61–80%) for enzymatically hydrolyzed *Wolffia globosa* [39] incubated at 80 °C for 10 min; different treatments have an impact on the stability, as the ultrasound-assisted extraction of protein and peptides from the same species portrayed an equivalent emulsion stability (98%) [34] to the values in this work after a 24 h incubation. Protein unfolding, generated by the ultrasound-mediated disruption of hydrophobic interactions and hydrogen bonds, was associated with improvements in the emulsion capacity [26]. Additionally, protein unfolding promotes more surface interactions, which, in combination with the presence of carbohydrates, can achieve more emulsifying stability. Apart from contribution of the reduction in the size of the aggregates, more solubility was also expected in the ultrasound sample due to the exposure of functional groups, which simultaneously enhanced protein migration into the emulsion interface, which results in greater stability [34].

## 3. Materials and Methods

### 3.1. Raw Material and Proximate Analysis

Sun-dried duckweed from Tlacotalpan (Veracruz, Mexico) was provided as leaf by the company Tecnopez and ground in a coffee grinder (Krups, Millville, NJ, USA) until a fine flour was obtained. The protein content and crude fat were determined by Microkjeldahl (AOAC 984.13-1994) [54] and Goldfish [55] protocols, respectively, whereas moisture and ashes were gravimetrically quantified according to official methods AOAC 925.10 and AOAC 923.03 (1992), respectively [56,57].

### 3.2. Optimization of the Alkaline Extraction of Protein Assisted with Ultrasound through a Box-Behnken Design of Experiments (DOE)

A Box–Behnken design of experiments was proposed to obtain the optimal condition for the alkaline extraction of protein from the duckweed flour through the modification of 3 factors at 3 levels. For this purpose, fifteen experiments were performed in duplicate by modifying the pH (9–14) of a duckweed flour dispersion in distilled water (1:10 *w*/*v*), followed by an ultrasound treatment in an UP400S ultrasound processor (Hielscher, Teltow, Germany), in which the ultrasound amplitude (20–100%) and time (10–30 min in 5 min slots) were modified as optimization factors. All the factor combinations are indicated in Table 2. Each extraction was followed by a one-hour incubation (72 RPM, 52 °C) and centrifugation at 4000 RPM (25 min, 23 °C). The supernatant volume was recorded, and the protein content (%) and content of solids (%) of 3 mL of supernatant were determined by MicroKjeldahl (AOAC 984.13-1994), and the gravimetric method (AOAC 925.10, weight after drying at 105 °C for 24 h), respectively.

With the obtained data, the design responses were the protein content (g protein/100 g dry extract), along with the protein extraction yield (PEY, %) and solids yield (SY, %), determined according to Equations (1) and (2).

(1) $$PEY\left(\%\right)=\frac{g\;of\;protein\;in\;the\;supernatant}{g\;of\;protein\;in\;the\;initial\;sample}\times 100$$

(2) $$SY\left(\%\right)=\frac{g\;of\;solids\;in\;the\;supernatant}{g\;of\;solids\;in\;the\;initial\;sample}\times 100$$

### 3.3. Comparison of the Effect of Ultrasound in the Alkaline Extraction of Protein

The optimized extraction point (pH = 11.5, US amplitude = 60%, and US time = 20 min) was carried out according to Section 3.2 until the centrifugation step, which was set at 10,000 RPM (10 min, 23 °C). The supernatant was collected, and the pH was modified to 4.5 with hydrochloric acid (HCl). The solution was centrifuged (10,000 RPM, 10 min, and 23 °C), and the pellet was freeze-dried for three days. This sample was referred to as the ultrasound sample. Another sample was extracted by following the same steps without the use of ultrasound, and this was referred to as the control sample.

#### 3.3.1. Colorimetric Analysis of the Samples

The CIELAB space was determined in the solid materials with a WR-10 precise color reader (FRU, Shenzhen, China) to obtain the L*, a*, and b* values.

#### 3.3.2. Scanning Electron Microscopy (SEM) of the Samples

The morphology of dry duckweed flour, as well as the freeze-dried alkaline protein extract and freeze-dried ultrasound-assisted protein extract, were obtained by coating a small sample amount with gold with a Q150R Rotatory Pump Coater (Quorum, London, UK), and imaging with an EVO M25 Scanning Electron Microscope (Carl Zeiss, Ciudad de Mexico, Mexico).

#### 3.3.3. Fourier Transformed Infrared Spectroscopy (FTIR) of the Samples

An FTIR analysis was performed in a Frontier FTIR Spectrometer (PerkinElmer, Waltham, MA, USA) using the transmittance mode for a spectral range of 4000 to 400 cm^−1^.

#### 3.3.4. Sodium Dodecyl Sulfate-Polyacrylamide Gel Electrophoresis (SDS-PAGE)

Both the ultrasound and control samples were dialyzed in double-distilled water for 72 h. The SDS-PAGE of each sample was then performed on a 15% gel with a discontinuous buffer system, as described by Laemmli [58]. Briefly, 3% protein dispersions were prepared in water at a pH of 7. The soluble fraction of the dispersion was mixed with a loading buffer in a 1:1 ratio, and 10 μL was loaded in the gel well. The electrophoresis was run at 70 V in stacking gel and at 90 V in separating gel until the tracking dye reached the bottom of the gel. Molecular weight standards of 15 to 250 KDa were simultaneously run with the samples (Bio-Rad Laboratories, Hercules, CA, USA). The gel was stained in 0.25% Coomassie brilliant blue R-250, distained in a solution containing 10% acetic acid and 45% ethanol, and scanned on an Image Scanner III (GE Healthcare, Amersham, UK).

#### 3.3.5. Functional Properties of the Obtained Samples

##### Water Solubility Index (%) and Water Absorption Index

A total of 1 g of the sample was mixed with 15 mL of distilled water in a 50 mL tube and vortexed until homogenization. The mixture was left at room temperature (RT) for 30 min and centrifuged at 5000 RPM for 20 min. The supernatant was collected, and its solids were gravimetrically obtained through incubation in a previously weighed tray at 100 °C for at least 4 h. The pellet was weighed, and the water solubility index and water absorption index were calculated according to Equations (3) and (4), respectively [59,60].

(3) $$Water\;Solubility\;Index\;\left(\%\right)=\frac{weigh\;of\;solids\;in\;supernatant\;}{initial\;sample\;weight}\times 100$$

(4) $$Water\;Absorption\;Index\;\left(\%\right)=\frac{pellet\;weight\;}{initial\;sample\;weight}$$

##### Foaming Activity (%), Foam Density (%), and Foam Stability (%)

A 3% protein dispersion (30 mL) was prepared in water, according to the protein content of each sample. The dispersion was agitated for 30 min at RT, and the foam and liquid volumes were recorded (F0, L0). The dispersion was then mixed with a classic hand mixer (Oster, Todos Santos, Mexico) at a speed of 5 for 13 min. The total foam (F1) and liquid (L1) volumes were immediately recorded, as well as the same volumes (F2, L2) after 15 min [61]. The foaming activity, foam density, and foam stability were calculated according to Equations (5)–(7), respectively.

(5) $$Foaming\;Activity\;\left(\%\right)=\left(\frac{L1+F1\;}{L0}-1\right)\times 100$$

(6) $$Foaming\;Activity\;\left(\%\right)=(\frac{L0-L1}{F1})\times 100$$

(7) $$Foaming\;Stability\;\left(\%\right)=(\frac{F1\;}{F2})\times 100$$

##### Emulsifying Capacity and Emulsifying Stability

A sample dispersion was prepared by mixing 0.7 g of the sample into 10 mL of distilled water for 30 min at RT. After vortexing in a tube, 10 mL of pure soybean oil (Nutrioli) was added, and the emulsion was formed with an T25 digital ultra-turrax (IKA, Staufen im Breisgau, Germany) for 2 min at 14,200 min^−1^. The formed emulsions are displayed in Figure S4. The total volume (*Vi*), oil volume (*Oi*), and emulsion volume (*Ei*) were immediately recorded, and the emulsions were left at 60 °C for 24 h, during which the total (*Vf*), oil (*Of*), and emulsion (*Ef*) volumes were recorded for stability [62]. The emulsion capacity and stability were calculated according to Equations (8) and (9), respectively.

(8) $$Emulsion\;capacity\;\left(\%\right)=(\frac{Ei}{Vi})\times 100$$

(9) $$Emulsion\;stability\;\left(\%\right)=[1-\left(\frac{Of}{Ef}\right)]\times 100$$

### 3.4. DOE Analysis and Statistical Analysis

The determination of the optimal extraction point, surface responses, predicted extraction values, and DOE equations and validation, as well as the statistical data analysis, were performed in Minitab 19.2020.1.0.

## 4. Conclusions

Duckweed (*Lemna minor*) has the potential to be applied to human consumption; for this reason, the optimization of the extraction of several compounds and molecules of interest is necessary. In the case of the integration of ultrasound during the alkaline extraction of proteins, pH, time, and amplitude were crucial for the optimization of different extraction parameters such as protein content and extract yields. The optimal point was determined at a pH of 11.5, an ultrasound amplitude of 60%, and an ultrasound time of 20 min. With that optimal condition, a two-fold increment was observed in the protein content in comparison to the initial duckweed flour. The cellular disruption produced by ultrasound was beneficial for the extraction of more protein, with a resulting improvement in its solubility, foaming activity, emulsifying capacity, and emulsion stability. These results are promising when considering the utilization of duckweed in diverse food products; however, special attention should be focused on the exploration of the safety, nutritional, and toxicological aspects of this plant. As duckweed was firstly explored for bioremediation and bioenergy production, many reports on its possible amount of toxins, pesticides, heavy metals, and antinutrients set the scenario for exploring the controlled production of duckweed if the main goal for this plant is its integration in the human diet. Nevertheless, duckweed is a promising alternative for the development of accessible and affordable food products.

## Figures and Tables

**Table 1:** Proximal analysis of duckweed (*Lemna minor*) and comparison with previous research.

Duckweed Specie *	Moisture (%, WB)	Fat (%, DW)	Protein (%, DW)	Ash (%, DW)	Total Carbohydrates ** (%, DW)	Reference
*Lemna minor*	6.23 ± 0.13	1.32 ± 0.10	21.74 ± 0.31	14.71 ± 0.08	ND	This work
*Lemna minor*	NR	4–4.4	16–45	4–5	NR	[16]
*Lemna gibba* (FD)	93.7 (F)	3.4	33.6	18	28.8	[29]
*Lemna gibba* L. (B)	10.03	7.61	22.72	13.23	36.23	[30]
*Wolffia arrhizal* (F)	95.18	6.07	50.89	11.71	31.3	[31]
*Spirodela polyrhiza* (F)	NR	7.11	35.82	18.51	38.38	[32]
Duckweed (DD)	3.7	NR	35.8	6.2	47.2	[33]
*Wolffia globosa* (D)	8.61	3.03	33.16	14.58	49.22	[34]
*Lemna minor*	NR	4.75	28.48	10.1	NR	[35]
*Lemna* spp.	NR	4.5–9.8	36.0–38.6	8.46–19.0	NR	[35]
Duckweed (SD)	NR	2.23	27.67	12.63	NR	[36]

Experimental data provided as mean ± standard deviation (SD); n = 3; * B = biomass; D = dried; DD = defatted and dried; F = fresh; FD = freeze-dried; SD = sun-dried; ** NR = not reported; ND = not determined; DW = dry weight; WB = wet basis.

**Table 2:** Factors and responses for the Box–Behnken experimental design of the alkaline extraction of duckweed protein assisted with ultrasound.

pH	Amplitude (%)	Time (min)	SY ^1^ (%)	PC ^1^ (%)	PEY ^1^ (%)
14.0	60	10	85.08 ± 7.99	14.60 ± 0.66	61.03 ± 8.47
14.0	100	20	79.62 ± 7.48	13.63 ± 0.31	54.88 ± 6.23
11.5	60	20	13.91 ± 4.20	38.73 ± 1.39	26.26 ± 7.03
9.0	60	30	15.06 ± 4.01	37.28 ± 6.37	26.90 ± 2.62
11.5	20	10	16.47 ± 1.32	35.74 ± 2.49	28.92 ± 4.33
14.0	20	20	73.69 ± 11.67	21.90 ± 8.90	41.46 ± 12.17
11.5	20	30	13.64 ± 2.39	38.44 ± 3.97	25.47 ± 1.84
11.5	100	10	17.74 ± 2.53	36.97 ± 3.69	31.91 ± 1.37
9.0	100	20	12.42 ± 1.67	36.79 ± 6.08	22.14 ± 0.70
11.5	100	30	16.05 ± 2.76	42.17 ± 1.84	33.30 ± 7.15
14.0	60	30	46.14 ± 5.11	21.22 ± 0.12	47.98 ± 5.04
11.5	60	20	22.08 ± 4.99	31.96 ± 2.05	34.34 ± 5.59
9.0	60	10	13.47 ± 2.34	36.70 ± 0.47	24.25 ± 4.52
9.0	20	20	13.74 ± 1.13	39.31 ± 3.40	24.89 ± 2.09
11.5	60	20	16.54 ± 2.05	40.72 ± 3.39	32.85 ± 1.34

^1^ SY = solids yield; PC = protein content (g protein/100 g of dry extract); PEY = protein extraction yield. Experimental data provided as mean ± SD; n = 2.

**Table 3:** Mathematical models for the three optimized responses.

Response	Regression Equation	r^2^
Solids Yield (%)	= 348.3 − 75.92 pH − 0.324 Amplitude + 5.39 Time + 4.106 pH × pH + 0.00106 Amplitude × Amplitude − 0.0323 Time × Time + 0.0181 pH × Amplitude − 0.4054 pH × Time + 0.00071 Amplitude × Time	95.77
Protein Content (%)	= −123.8 + 32.72 pH + 0.054 Amplitude − 0.747 Time − 1.609 pH × pH + 0.000516 Amplitude × Amplitude + 0.0037 Time × Time − 0.0144 pH × Amplitude + 0.0604 pH × Time + 0.00156 Amplitude × Time	86.66
Protein Yield (%)	= 120.8 − 21.22 pH − 0.254 Amplitude + 0.88 Time + 1.187 pH × pH − 0.00170 Amplitude × Amplitude + 0.0148 Time × Time + 0.0404 pH × Amplitude − 0.1570 pH × Time + 0.00302 Amplitude × Time	86.65

**Table 4:** Validation of the optimal point for the Box–Behnken experimental design between real and predicted values.

Condition	pH	Amplitude	Time		SY (%)	PC (%)	PEY (%)
Optimal Point	11.5	60	20	Predicted	17.51	37.14	31.15
				Real	14.86 ± 0.26	41.30 ± 1.24	30.08 ± 0.90
				%Fit	84.87	111.20	96.57

**Table 5:** CIELAB parameters of color for duckweed and its protein extracts.

Sample	L*	a*	b*	Appearance	RGB Space
Duckweed Flour	47.32 ± 0.75 ^a^	4.04 ± 0.13 ^b^	35.60 ± 0.45 ^c^	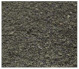	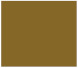
Control Protein Extract	45.66 ± 1.32 ^a^	4.51 ± 0.10 ^b^	39.06 ± 0.11 ^b^	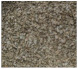	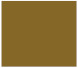
Ultrasound Protein Extract	24.41 ± 0.48 ^b^	9.64 ± 0.05 ^a^	79.04 ± 0.64 ^a^	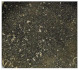	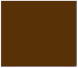

Experimental data provided as mean ± standard deviation (SD); n = 3. Different letters within the same column indicate significant differences (*p* < 0.05).

## Data Availability

Data are contained within the article and Supplementary Materials.

## Supplementary Materials

The following supporting information can be downloaded at https://www.mdpi.com/article/10.3390/molecules29051122/s1. Figure S1: Analysis of variance for the response surface regression of the extract yield; Table S1: Analysis of variance for the response surface regression of the extract yield; Figure S2: Pareto chart of the standardized effects on the protein content; Table S2. Analysis of variance for the response surface regression of the protein content; Figure S3: Pareto chart of the standardized effects on the protein yield; Table S3. Analysis of variance for the response surface regression of the protein yield; Figure S4: Emulsions produced with (a) duckweed, (b) control protein extract, and (c) ultrasound protein extract.
