# Supplementary material for: The Effect of Ultrasound on the Extraction and Functionality of Proteins from Duckweed (Lemna minor)
Source: Molecules. 2024 Mar 1;29(5):1122. doi: 10.3390/molecules29051122 (PMC10935346; doi:10.3390/molecules29051122)
Supplement: Supplementary file 1 [file molecules-29-01122-s001.zip › molecules-2843346-supplementary.pdf]

Supplementary Material

for

The Effect of Ultrasound on the Extraction and Functionality of Proteins from Duckweed (*Lemna minor*)

Vicente Antonio Mirón-Mérida [1], Cintya Soria-Hernández [1,*], Alejandro Richards-Chávez [1], Juan Carlos Ochoa-García [1], Jorge Luis Rodríguez-López [1] and Cristina Chuck-Hernández [2,*]

[1] Escuela de Ingeniería y Ciencias, Tecnologico de Monterrey, Av. Eugenio Garza Sada 2501, Colonia Tecnológico, Monterrey 64700, Mexico

[2] Instituto para la Investigación en Obesidad, Tecnologico de Monterrey, Av. Eugenio Garza Sada 2501, Sur Tecnológico, Monterrey 64849, Mexico

* Correspondence: cintya.soria@tec.mx (C.S.-H.); cristina.chuck@tec.mx (C.C.-H.)

Content

1. Table S1. Analysis of Variance for the response surface regression of the extract yield.
2. Figure S1. Pareto chart o the standardized effects on the extract yield.
3. Table S2. Analysis of Variance for the response surface regression of the protein content.
4. Figure S2. Pareto chart o the standardized effects on the protein content.
5. Table S3. Analysis of Variance for the response surface regression of the protein yield.
6. Figure S3. Pareto chart o the standardized effects on the protein yield.
7. Figure S4. Emulsions produced with (a) duckweed, (b) control protein extract, and (c) ultrasound protein extract.

Table S1. Analysis of Variance for the response surface regression of the extract yield.

Source	DF	Adj SS	Adj MS	F-Value	P-Value
Model	9	19575.2	2175.0	50.32	0.000
Linear	3	13662.4	4554.1	105.36	0.000
pH	1	13207.2	13207.2	305.54	0.000
Amplitude	1	17.2	17.2	0.40	0.535
Time	1	438.1	438.1	10.13	0.005
Square	3	5064.3	1688.1	39.05	0.000
pH*pH	1	4862.9	4862.9	112.50	0.000
Amplitude*Amplitude	1	21.3	21.3	0.49	0.491
Time*Time	1	77.1	77.1	1.78	0.197
2-Way Interaction	3	848.5	282.8	6.54	0.003
pH*Amplitude	1	26.3	26.3	0.61	0.445
pH*Time	1	821.5	821.5	19.01	0.000
Amplitude*Time	1	0.6	0.6	0.01	0.904
Error	20	864.5	43.2		
Lack-of-Fit	3	419.2	139.7	5.33	0.009
Pure Error	17	445.3	26.2		
Total	29	20439.8			

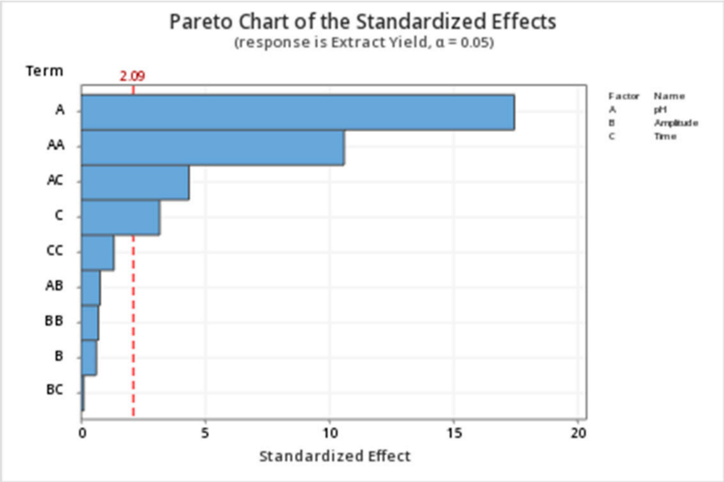

Figure S1. Pareto chart o the standardized effects on the extract yield.

Table S2. Analysis of Variance for the response surface regression of the protein content.

Source	DF	Adj SS	Adj MS	F-Value	P-Value
Model	9	2425.32	269.48	14.43	0.000
Linear	3	1614.21	538.07	28.82	0.000
pH	1	1548.62	1548.62	82.93	0.000
Amplitude	1	8.51	8.51	0.46	0.507
Time	1	57.08	57.08	3.06	0.096
Square	3	773.21	257.74	13.80	0.000
pH*pH	1	746.33	746.33	39.97	0.000
Amplitude*Amplitude	1	5.02	5.02	0.27	0.610
Time*Time	1	1.01	1.01	0.05	0.818
2-Way Interaction	3	37.90	12.63	0.68	0.577
pH*Amplitude	1	16.56	16.56	0.89	0.358
pH*Time	1	18.24	18.24	0.98	0.335
Amplitude*Time	1	3.10	3.10	0.17	0.688
Error	20	373.46	18.67		
Lack-of-Fit	3	63.63	21.21	1.16	0.353
Pure Error	17	309.83	18.23		
Total	29	2798.77			

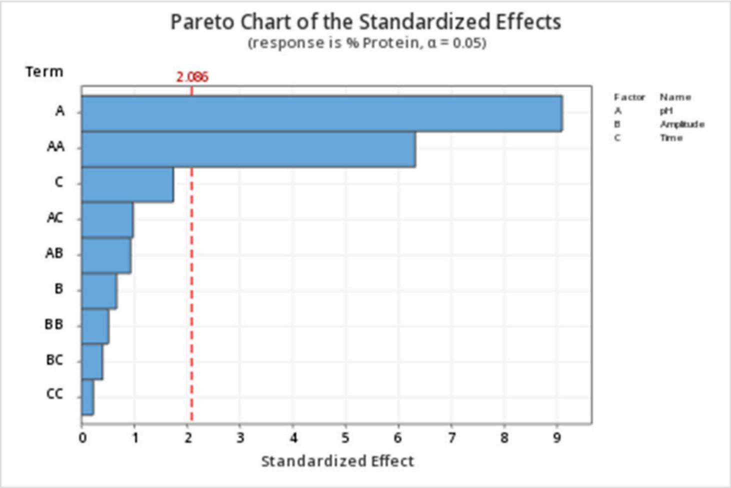

Figure S2. Pareto chart o the standardized effects on the protein content.

Table S3. Analysis of Variance for the response surface regression of the protein yield.

Source	DF	Adj SS	Adj MS	F-Value	P-Value
Model	9	3787.97	420.89	14.43	0.000
Linear	3	3025.84	1008.61	34.57	0.000
pH	1	2871.43	2871.43	98.42	0.000
Amplitude	1	115.55	115.55	3.96	0.060
Time	1	38.86	38.86	1.33	0.262
Square	3	496.47	165.49	5.67	0.006
pH*pH	1	406.26	406.26	13.93	0.001
Amplitude*Amplitude	1	54.67	54.67	1.87	0.186
Time*Time	1	16.11	16.11	0.55	0.466
2-Way Interaction	3	265.66	88.55	3.04	0.053
pH*Amplitude	1	130.74	130.74	4.48	0.047
pH*Time	1	123.23	123.23	4.22	0.053
Amplitude*Time	1	11.70	11.70	0.40	0.534
Error	20	583.49	29.17		
Lack-of-Fit	3	35.68	11.89	0.37	0.776
Pure Error	17	547.81	32.22		
Total	29	4371.45			

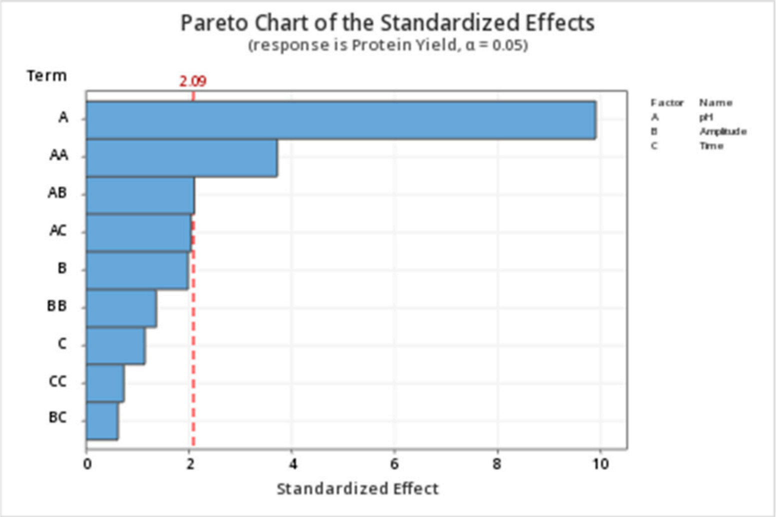

Figure S3 . Pareto chart o the standardized effects on the protein yield.

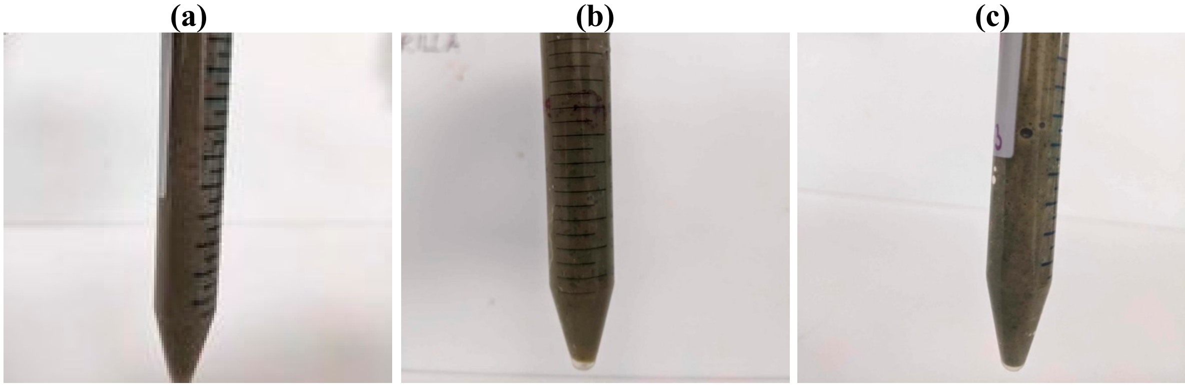

Figure S4. Emulsions produced with **(a)** duckweed flour, **(b)** control protein extract, and **(c)** ultrasound protein extract.
